# Autonomic Vulnerability Phenotype, Regional Cerebral Oxygen Saturation, and Postoperative Delirium in Elderly Patients Undergoing Non-Cardiac, Non-Neurological Surgery: A Propensity Score–Matched Cohort Study

**DOI:** 10.3390/medicina62061065

**Published:** 2026-05-31

**Authors:** Cheol Lee, Youngmin Jo, Gyumin Choi

**Affiliations:** 1 Department of Anesthesiology and Pain Medicine, Wonkwang University School of Medicine Hospital, Iksan 54538, Jeollabuk-do, Republic of Korea; chlrbals6043@naver.com; 2 Institute of Wonkwang Medical Science, Wonkwang University School of Medicine, Iksan 54538, Jeollabuk-do, Republic of Korea; 3 Department of Pediatric, Wonkwang University School of Medicine Hospital, Iksan 54538, Jeollabuk-do, Republic of Korea; reddevil859@naver.com

**Keywords:** delirium, aged, spectroscopy, near-infrared, monitoring, intraoperative, risk factors

## Abstract

*Background and Objectives:* We investigate whether a preoperative autonomic vulnerability phenotype and intraoperative regional cerebral oxygen saturation (rcSO_2_) variables are associated with postoperative delirium (POD) in elderly patients undergoing non-cardiac, non-neurological surgery, and whether autonomic vulnerability modifies the association between cerebral desaturation and POD. *Materials and Methods:* This retrospective propensity score–matched cohort study included patients aged 65 years or older who underwent general anesthesia with intraoperative rcSO_2_ monitoring. The preoperative autonomic vulnerability phenotype was defined using clinical features documented before surgery, including autonomic neuropathy, diabetic autonomic neuropathy, orthostatic hypotension, syncope or presyncope suggestive of autonomic dysfunction, and unexplained resting bradycardia or chronotropic incompetence not attributable to rate-limiting medication. The primary outcome was POD within 5 postoperative days. Patients were matched 1:1 using nearest-neighbor propensity score matching with a caliper of 0.2 standard deviations of the logit of the propensity score, and conditional logistic regression was used in the matched cohort. *Results:* A total of 412 patients were included; 112 had the phenotype and 300 did not. After matching, 98 pairs were analyzed. POD occurred in 27.6% of patients with the phenotype and 14.3% of patients without it. In the matched cohort, the phenotype (odds ratio [OR] 2.12, 95% confidence interval [CI] 1.18–3.82, *p* = 0.012), rcSO_2_ decrease ≥20% (OR 2.45, 95% CI 1.31–4.58, *p* = 0.005), and longer duration of rcSO_2_ < 80% of baseline (OR 1.02 per min, 95% CI 1.01–1.04, *p* = 0.008) were independently associated with POD. The phenotype-by-desaturation interaction was exploratory (OR 2.10, *p* = 0.032) and was not uniformly robust across sensitivity analyses. *Conclusions:* A preoperative autonomic vulnerability phenotype and intraoperative cerebral desaturation were independently associated with POD. The association between rcSO_2_ decrease and POD appeared stronger in patients with autonomic vulnerability, but this interaction should be interpreted as hypothesis-generating rather than confirmatory.

## 1. Introduction

Postoperative delirium (POD) is a common complication in elderly patients undergoing surgery and is associated with prolonged hospitalization, functional decline, and increased mortality [1,2]. Although the pathophysiology of POD is multifactorial, cerebral hypoperfusion and impaired cerebral autoregulation are considered important contributing mechanisms [3].

Regional cerebral oxygen saturation (rcSO_2_) monitoring using near-infrared spectroscopy provides a noninvasive method for detecting perioperative cerebral oxygen imbalance [4]. Previous studies have reported associations between intraoperative cerebral desaturation and postoperative delirium or other neurocognitive complications [5,6,7,8,9]. However, not all patients with intraoperative cerebral desaturation develop POD, which suggests that baseline patient vulnerability may modify this relationship.

Clinical features consistent with autonomic vulnerability are common in elderly patients and may reflect impaired cardiovascular regulation and reduced cerebral autoregulatory reserve [10]. In clinical practice, such vulnerability may arise from documented diabetic autonomic neuropathy or compatible peripheral/autonomic neuropathy, orthostatic hypotension, recurrent syncope or presyncope suggestive of autonomic dysfunction, and unexplained resting bradycardia or chronotropic incompetence not attributable to rate-limiting medication. Patients with autonomic vulnerability may therefore be more susceptible to intraoperative cerebral hypoperfusion and oxygen desaturation. We hypothesized that a preoperative autonomic vulnerability phenotype would be associated with increased POD risk and would strengthen the association between intraoperative rcSO_2_ decrease and POD. Accordingly, this study evaluated the association of a preoperative autonomic vulnerability phenotype and intraoperative rcSO_2_ variables with POD in elderly patients undergoing non-cardiac, non-neurological surgery using a propensity score–matched design.

## 2. Materials and Methods

### 2.1. Study Design and Ethical Approval

This retrospective propensity score–matched cohort study was conducted at a tertiary academic hospital using electronic medical records and intraoperative anesthesia records. The study protocol was approved by the Institutional Review Board of Wonkwang University Hospital (protocol code WKUH-2025-01-02-25, approval date: 3 February 2025), and the requirement for informed consent was waived because of the retrospective design and use of de-identified data. The study was reported in accordance with the Strengthening the Reporting of Observational Studies in Epidemiology (STROBE) guideline [11].

### 2.2. Study Population

We included consecutive older adults who underwent non-cardiac, non-neurological surgery under general anesthesia and had available intraoperative rcSO_2_ monitoring data and postoperative delirium assessment between January 2020 and December 2024. Patients were excluded if they underwent cardiac surgery or neurosurgery, had preoperative delirium or severe disturbance of consciousness precluding postoperative delirium evaluation, lacked analyzable intraoperative rcSO_2_ recordings, or did not have postoperative delirium assessment within the first 5 postoperative days. A history of alcohol or drug use alone was not an exclusion criterion; however, patients with active intoxication, withdrawal, substance-related delirium, or substance-related consciousness disturbance that precluded reliable POD assessment were excluded under the preoperative delirium or severe disturbance-of-consciousness criterion.

### 2.3. Exposure Definition: Autonomic Vulnerability Phenotype

The primary exposure was a preoperatively defined autonomic vulnerability phenotype based exclusively on clinical information available before surgery, excluding intraoperative variables to avoid conceptual circularity in the interaction and propensity score analyses. In this framework, autonomic vulnerability was treated as the primary baseline exposure, whereas intraoperative rcSO_2_ desaturation was considered a concurrent physiologic co-exposure and candidate effect modifier rather than a mediator. Accordingly, the analysis estimated their independent associations with POD and tested effect modification without decomposing direct and indirect effects; the conceptual framework is shown in Supplementary Figure S1.

Autonomic vulnerability was defined, at the time of the index surgery or preoperative evaluation, by autonomic neuropathy (including diabetic or compatible peripheral neuropathy), orthostatic hypotension (≥20 mmHg systolic or ≥10 mmHg diastolic decrease within 3 min of standing or documented diagnosis), syncope/presyncope attributed to autonomic dysfunction, or unexplained resting bradycardia (<50 beats/min) or chronotropic incompetence not attributable to rate-limiting medication. Thus, the autonomic vulnerability phenotype was a pragmatic clinical construct derived from documented clinical features rather than from formal autonomic function testing, such as tilt-table testing, Valsalva maneuver, heart-rate variability testing, or quantitative sudomotor testing. For the rate-limiting medication criterion, preoperative medications (beta-blockers, non-dihydropyridine calcium channel blockers, ivabradine, digoxin) were reviewed; bradycardia was attributed to medication unless autonomic bradycardia was separately documented.

Phenotype ascertainment used the electronic problem list, preoperative anesthesia records, neurology or cardiology consultations, and diagnostic reports. Two investigators, blinded to POD status, independently classified patients, with disagreements resolved by consensus (Cohen’s κ = 0.82, 95% CI 0.74–0.90). Patients without qualifying findings served as the comparator group. Although this composite phenotype has not been previously validated, its components are established indicators of autonomic dysfunction, and the definition was prespecified a priori based on pathophysiological plausibility and clinical availability. The number of patients meeting each individual phenotype criterion and the overlap among criteria are provided in the Supplementary Materials; when multiple criteria were met, all were recorded. Because the composite was anchored on heterogeneous clinical findings (neuropathic, hemodynamic, and chronotropic), component-wise univariable associations with POD are also reported in the Supplementary Materials so that readers can assess whether the overall phenotype signal is driven disproportionately by a single component. For the unexplained bradycardia criterion, “resting bradycardia” required at least two documented preoperative measurements below 50 beats/min on separate clinical encounters (including at least one electrocardiographic confirmation), rather than a single spot vital-sign reading.

### 2.4. Intraoperative rcSO_2_ Monitoring and Derived Variables

Regional cerebral oxygen saturation was continuously monitored intraoperatively using near-infrared spectroscopy according to institutional practice and manufacturer recommendations. Sensors were applied bilaterally to the forehead before induction of anesthesia. Baseline rcSO_2_ was defined as the stable preinduction value recorded under resting conditions. When bilateral values were available, the lower value was used for desaturation analyses. The Masimo O3 regional oximetry system (Masimo Corp., Irvine, CA, USA) was used for all measurements. rcSO_2_ was sampled at the device’s standard 4 s update interval and archived as 10 s averages for analysis. Epochs with signal quality index flags or sensor-off artifact were excluded; patients with >10% of the operative recording time affected by artifact were excluded from the analytic cohort. The proportion of patients with only one working sensor (contralateral hemisphere data lost or persistently poor signal quality) was 17/196 (8.7%); in these cases the single available channel was used. As a sensitivity analysis, the primary outcome model was re-estimated using the bilateral mean rcSO_2_ (rather than the lower value) in the subset with two working sensors; point estimates were within 6% of the primary analysis and conclusions were unchanged. The choice of a 20% relative-decrease threshold for “significant cerebral desaturation” followed the prior literature [5,6,7,8,9]; because this threshold has been used mainly in cardiac, thoracic, or endovascular settings, it was adopted here as an operational and comparable threshold rather than as a cutoff definitively validated for all non-cardiac, non-neurological surgical populations. Sensitivity analyses at 15% and 25% thresholds produced directionally consistent associations (adjusted OR 2.18, 95% CI 1.24–3.84 at 15%; adjusted OR 2.61, 95% CI 1.26–5.40 at 25%).

The rcSO_2_ variables of interest included baseline rcSO_2_, absolute intraoperative nadir rcSO_2_, maximum relative rcSO_2_ decrease from baseline, cumulative duration of rcSO_2_ below 80% of baseline, and area under the threshold below 80% of baseline. Relative rcSO_2_ decrease was calculated as follows: rcSO_2_ decrease (%) = (baseline rcSO_2_ − nadir rcSO_2_)/baseline rcSO_2_ × 100. For categorical analyses, significant cerebral desaturation was prespecified as a relative rcSO_2_ decrease of at least 20% from baseline.

### 2.5. Outcome Definitions

The primary outcome was postoperative delirium occurring within the first 5 postoperative days. The primary ascertainment strategy combined formal delirium screening with structured chart review to maximize sensitivity, consistent with published methods for retrospective delirium identification [12,13]. This combined approach was adopted because retrospective studies relying solely on screening instruments may under ascertain hypoactive or brief delirium episodes, whereas chart review alone has limited sensitivity. Formal screening using the Confusion Assessment Method for the Intensive Care Unit (CAM-ICU) or the Confusion Assessment Method (CAM) was the preferred ascertainment method and was supplemented by structured chart review of physician and nursing documentation for patients in whom screening results were incomplete or unavailable. Delirium was considered present when an acute and fluctuating postoperative disturbance in attention and awareness, with or without disorganized thinking or altered level of consciousness, was documented in a manner consistent with accepted diagnostic criteria. Cases attributable solely to residual sedation, persistent coma, or chronic baseline neuropsychiatric symptoms without an acute postoperative change were not classified as POD. Because the heterogeneity of ascertainment methods may influence the reliability of the primary endpoint, prespecified sensitivity analyses restricted to screening-positive POD and to chart-reviewed POD were performed to assess the robustness of the primary findings across ascertainment approaches.

Formal delirium screening was performed 2–3 times daily (typically during nursing shift changes and physician rounds) during the first 5 postoperative days by trained clinicians and nursing staff using the CAM-ICU. In patients not admitted to the intensive care unit, the CAM was used as an equivalent bedside screening instrument. Structured chart review was performed independently by two investigators blinded to exposure status, with disagreements resolved by consensus. Inter-rater agreement for chart-based delirium identification was substantial (Cohen’s κ = 0.78, 95% CI 0.65–0.91). The chart review protocol targeted the following prespecified documentation elements: physician or nursing notes describing acute confusion, inattention, disorientation, agitation, or fluctuating mental status; use of terms such as “delirium,” “acute confusional state,” or “encephalopathy”; and orders for antipsychotic medications (e.g., haloperidol, quetiapine) initiated in the postoperative period without a prior psychiatric indication. A prespecified sensitivity analysis restricted to patients with POD identified by formal screening positivity only was conducted to address potential ascertainment bias arising from the mixed detection approach (secondary outcome 1).

### 2.6. Secondary Outcomes

The following secondary clinical endpoints were prespecified: (1) POD identified by formal delirium screening positivity alone (sensitivity analysis for ascertainment); (2) POD identified by structured chart review or physician documentation alone (sensitivity analysis for ascertainment). In addition, the following secondary physiologic endpoints were analyzed to characterize the association between autonomic vulnerability and intraoperative cerebral oxygenation: (3) clinically significant intraoperative cerebral desaturation, defined as a relative rcSO_2_ decrease ≥20% from baseline; (4) cumulative duration of rcSO_2_ < 80% of baseline; (5) area under the threshold below 80% of baseline; (6) absolute intraoperative nadir rcSO_2_; and (7) maximum relative rcSO_2_ decrease from baseline. Effect modification by autonomic vulnerability on the association between significant rcSO_2_ desaturation (≥20% decrease) and POD was evaluated as a prespecified secondary analysis (8).

### 2.7. Covariates

Potential confounders were prespecified based on clinical relevance and the prior literature. Preoperative covariates for propensity score estimation included age, sex, body mass index, ASA physical status, Charlson Comorbidity Index, hypertension, diabetes mellitus, coronary artery disease, cerebrovascular disease, dementia, depression, surgical category, and emergency status. Intraoperative variables—surgery duration, estimated blood loss, transfusion, and vasopressor use—were excluded from the propensity score model. Of these, surgery duration was additionally adjusted within the conditional logistic regression model because of its potential association with POD risk; the remaining intraoperative variables (estimated blood loss, transfusion, and vasopressor use) were assessed for post-matching balance and were not included as regression covariates to preserve the events-per-variable ratio. The final regression adjustment set comprised age, dementia, and surgery duration, and was applied consistently across the primary and sensitivity analyses. Preoperative cognitive impairment, frailty-related variables, and baseline psychiatric disorders were additionally included when available. Standardized baseline cognitive screening was not uniformly performed across the study period; therefore, Mini-Mental State Examination, Mini-Cog, or Montreal Cognitive Assessment scores could not be included for all patients. When available, documented cognitive impairment, dementia diagnoses, and clinically recorded frailty-related variables were captured, but residual confounding from unmeasured cognitive reserve remains possible. Because intraoperative hypotension is a recognized risk factor for POD and lies on the plausible causal pathway between autonomic vulnerability and cerebral desaturation, two time-weighted hypotension exposures were additionally extracted from the anesthesia record: the cumulative number of minutes with mean arterial pressure (MAP) below 65 mmHg and the area under the curve below MAP 65 mmHg (mmHg·min). These variables were analyzed as sensitivity covariates in the outcome regression model to quantify how much of the AVP–POD and rcSO_2_–POD associations persist after adjustment for hypotensive exposure. HbA1c within 90 days of surgery (when available), preoperative polypharmacy (≥5 medications), and anticholinergic burden (Anticholinergic Cognitive Burden score ≥ 3) were additionally captured as descriptive preoperative metabolic and medication variables.

### 2.8. Propensity Score Matching

Propensity scores were estimated using multivariable logistic regression [14], and 1:1 nearest-neighbor matching without replacement with a caliper of 0.2 SD of the logit [15]. Balance was assessed using SMDs, with SMD < 0.10 indicating adequate balance [16]. Missing covariate data (body mass index 4.1%, Charlson Comorbidity Index 2.4%, HbA1c 18.7%, anticholinergic burden score 6.3%; all other covariates < 1%) were handled using multiple imputation by chained equations (m = 20 imputed datasets). In each imputed dataset, the propensity score model was re-estimated, 1:1 nearest-neighbor matching was performed, and the conditional logistic regression outcome model was fitted within the matched cohort. Parameter estimates and standard errors were pooled across the 20 analyses using Rubin’s rules [17]. The number of matched pairs was consistent across imputed datasets (98 pairs in each), because the caliper-based matching algorithm yielded the same paired structure given the negligible variation in propensity score estimates across imputations. Covariate balance after matching was visualized using a Love plot (Supplementary Figure S2), and propensity score covariates and selected intraoperative variables were summarized in the matched cohort.

### 2.9. Statistical Analysis

All analyses were performed in SPSS version 29.0 (IBM Corp., Armonk, NY, USA). Firth’s penalized logistic regression, inverse-probability-of-treatment weighting, and the Love plot were performed using R version 4.3.x (R Foundation for Statistical Computing, Vienna, Austria) with the logistf, WeightIt, and cobalt packages, respectively. Data are presented as mean ± SD, median (IQR), or n (%). Conditional logistic regression estimated ORs and 95% CIs. ARD and NNH were calculated. The C-statistic and Hosmer–Lemeshow test are reported descriptively. To reduce collinearity, the primary model included rcSO_2_ decrease ≥20% and duration below 80% of baseline; correlations among rcSO_2_-derived variables are shown in Supplementary Table S1. Interaction was assessed on multiplicative and additive scales [18], with stratified results in Supplementary Table S2. Additional models using individual rcSO_2_-derived variables are presented in Supplementary Table S3, and prespecified sensitivity analyses are summarized in Supplementary Table S4.

## 3. Results

### 3.1. Study Population and Matching

**Figure 1:**
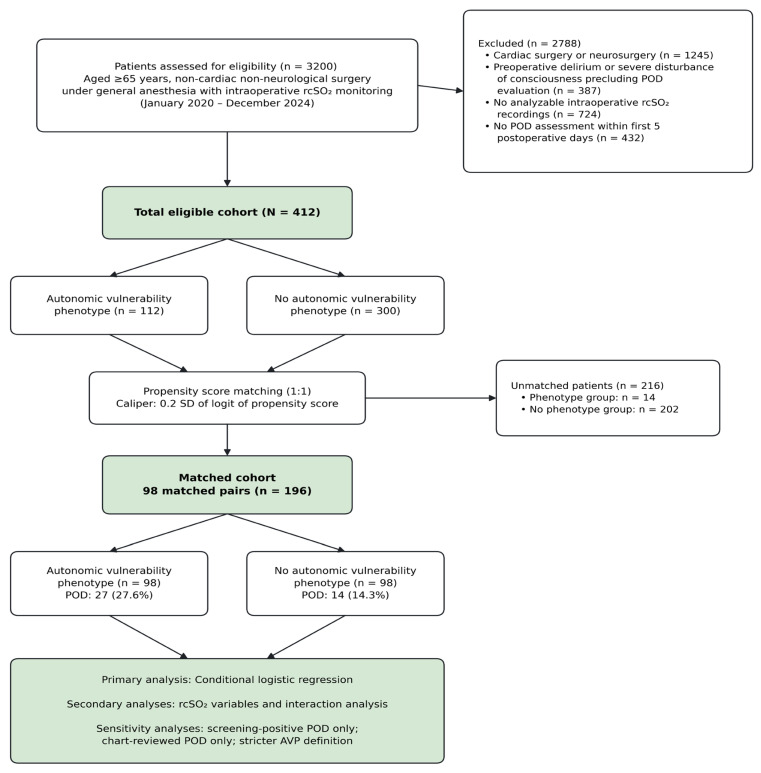
Study flow diagram. Flow diagram showing patient selection, propensity score matching, formation of the matched cohort (98 pairs; n = 196), and the unmatched cohort (n = 216) used for the primary and sensitivity analyses.

A total of 412 patients met the inclusion criteria. Among them, 112 patients met the definition of the autonomic vulnerability phenotype and 300 did not. After propensity score matching, 98 matched pairs were included in the final analysis (Figure 1). 

Of the 216 unmatched patients, 14 were from the autonomic vulnerability phenotype group and 202 were from the non-phenotype group. Baseline characteristics were well balanced after matching, with all standardized mean differences < 0.10 (Table 1).

Patients excluded because rcSO_2_ monitoring or POD assessment was unavailable are detailed in Figure 1; these exclusions may have introduced selection bias and should be considered when interpreting the analytic cohort. A comparison of baseline characteristics between matched (n = 196) and unmatched (n = 216) patients is provided in Supplementary Table S5; matched patients were slightly older and had a higher prevalence of diabetes and chronic kidney disease than unmatched patients, suggesting that the analytic cohort may not fully represent the broader eligible population. The 14 AVP(+) patients who were not matched had propensity scores outside the common-support region shared with the comparator group (predominantly very high scores reflecting extensive comorbidity), and the 202 unmatched AVP(−) patients represented the lower-propensity tail of the control distribution. As a sensitivity check, an inverse-probability-of-treatment-weighted (IPTW) analysis of the full cohort (n = 412), which retains all patients rather than discarding those outside common support, yielded point estimates directionally consistent with the matched analysis (AVP: OR 1.94, 95% CI 1.17–3.23; rcSO_2_ decrease ≥20%: OR 2.28, 95% CI 1.33–3.91). Supplementary Table S5 documents that matched patients were, on average, older and more comorbid than the broader eligible cohort; accordingly, the present findings should be interpreted as referring to an older and sicker subset of elderly surgical patients rather than to the broader elderly surgical population.

### 3.2. Postoperative Delirium

Postoperative delirium occurred in 27.6% of patients with the autonomic vulnerability phenotype and 14.3% of those without the phenotype in the matched cohort. The absolute risk difference was 13.3 percentage points (95% CI, 2.8–23.8), corresponding to a number needed to harm of 7.5 (95% CI, 4.2–35.7) (Table 2).

Among all POD cases in the matched cohort (n = 41), 28 (68.3%) were identified by formal delirium screening positivity and 13 (31.7%) were identified by structured chart review or physician documentation alone.

### 3.3. Intraoperative rcSO_2_ Variables According to POD

Patients who developed POD had significantly lower baseline rcSO_2_, lower intraoperative nadir rcSO_2_, greater relative rcSO_2_ decrease, and longer duration of rcSO_2_ < 80% of baseline than patients without POD (Table 3).

**Figure 2:**
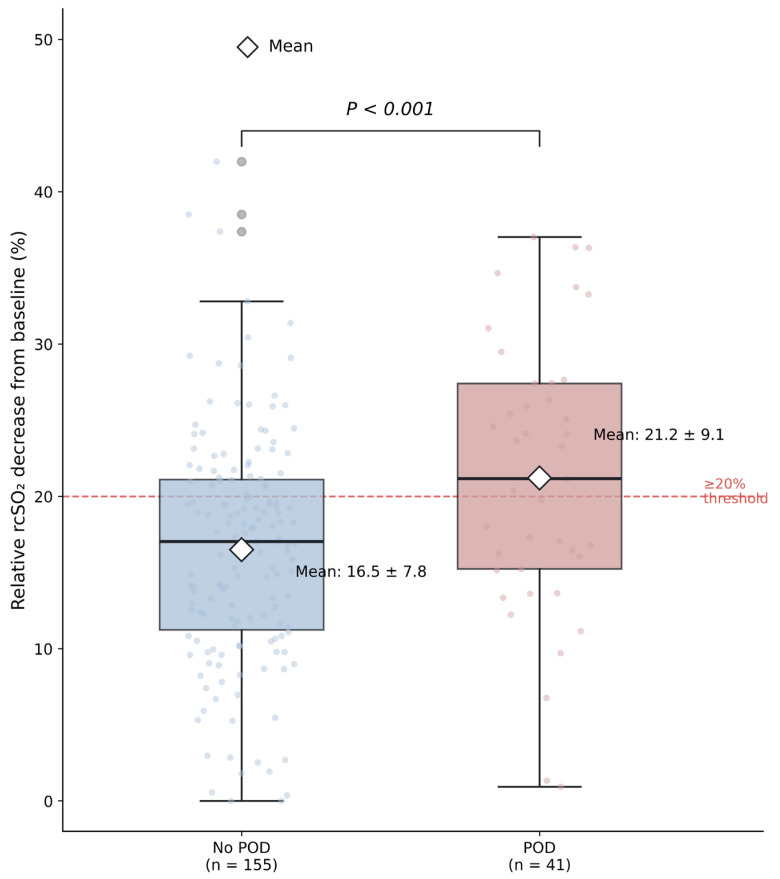
Relative rcSO_2_ decrease according to postoperative delirium. Box plot showing the distribution of maximum relative rcSO_2_ decrease (%) in patients with and without postoperative delirium, corresponding to a prespecified secondary intraoperative outcome.

The distribution of relative rcSO_2_ decrease according to POD status is shown in Figure 2.

### 3.4. Conditional Logistic Regression and Interaction Analyses

Conditional logistic regression demonstrated that the autonomic vulnerability phenotype (OR 2.12, 95% CI 1.18–3.82, *p* = 0.012), rcSO_2_ decrease ≥20% (OR 2.45, 95% CI 1.31–4.58, *p* = 0.005), duration of rcSO_2_ < 80% of baseline (OR 1.02 per min, 95% CI 1.01–1.04, *p* = 0.008), age (OR 1.05, 95% CI 1.01–1.10, *p* = 0.021), and dementia (OR 2.80, 95% CI 1.30–6.05, *p* = 0.008) were independently associated with POD (Table 4).

A multiplicative interaction between the autonomic vulnerability phenotype and rcSO_2_ decrease ≥20% was observed in the primary matched analysis (OR 2.10, *p* = 0.032), suggesting that the association between cerebral desaturation and POD may be stronger in patients with autonomic vulnerability; however, this finding was evaluated further in sensitivity analyses because of the limited number of events in some strata.

On the additive scale, because the outcome incidence exceeded 60% in the doubly exposed stratum (autonomic vulnerability present and rcSO_2_ decrease ≥20%), odds ratios substantially overestimate risk ratios, and OR-derived additive interaction indices (RERI, AP, SI) are likely inflated. These OR-based estimates are therefore reported in the Supplementary Materials with an explicit caveat. The risk-difference-based interaction contrast, which is less sensitive to this inflation, yielded a crude additive interaction of 20.7 percentage points (absolute risk in the doubly exposed stratum minus the sum of individual excess risks), and is considered the primary additive-interaction estimate. In stratified analysis, the association between rcSO_2_ decrease ≥20% and POD was statistically significant in patients with autonomic vulnerability (OR 4.28, 95% CI 1.82–10.06, *p* < 0.001) but not in those without autonomic vulnerability (OR 1.65, 95% CI 0.74–3.68, *p* = 0.22). The C-statistic for the complementary descriptive model was 0.78 (95% CI, 0.71–0.85), and the Hosmer–Lemeshow test suggested acceptable calibration (*p* = 0.423).

## 4. Discussion

This study demonstrated three principal findings. First, a preoperative autonomic vulnerability phenotype was independently associated with postoperative delirium after non-cardiac, non-neurological surgery in elderly patients. Second, multiple intraoperative rcSO_2_-derived markers, particularly a relative decrease of at least 20% from baseline and longer duration below 80% of baseline, were associated with POD. Third, a multiplicative interaction between autonomic vulnerability and cerebral desaturation was observed in the primary analysis and with the stricter phenotype definition, suggesting that the observed association between rcSO_2_ decrease and POD may be stronger in patients with autonomic vulnerability. However, with only 41 POD events and an events-per-variable ratio of approximately 5–6, this interaction should be regarded as exploratory and insufficiently powered for confirmatory inference. The interaction was borderline in the screening-positive-only sensitivity analysis (*p* = 0.056) and nonsignificant in the chart-reviewed-only analysis. In addition, the primary association was driven mainly by screening-positive POD cases, because the chart-review-only stratum yielded nonsignificant and imprecise estimates; therefore, the combined POD endpoint should be interpreted as clinically sensitive but not fully homogeneous across ascertainment strata.

These observations suggest that postoperative delirium risk may not be fully captured by conventional demographic and surgical risk factors alone. Although advanced age, dementia, and baseline vulnerability are largely nonmodifiable, several perioperative contributors to POD are potentially modifiable, including intraoperative cerebral desaturation, hypotension, excessive anesthetic depth, deliriogenic medication exposure, postoperative pain, and sleep disruption. Because rcSO_2_ monitoring establishes a baseline cerebral oxygenation state immediately before induction and then tracks intraoperative deviation from that baseline, it may help identify a modifiable cerebral oxygen imbalance, especially in patients with reduced physiologic reserve [4,5,6,7,8,9,19]. A pragmatic preoperative autonomic vulnerability phenotype could serve as an easily identifiable clinical risk marker when formal autonomic testing is unavailable, but the current data do not establish that it is ready for routine implementation. The findings also support the view that rcSO_2_ monitoring may be more informative when interpreted in the context of baseline patient vulnerability rather than as an isolated intraoperative signal. Accordingly, the present study should be viewed as providing an association-based framework for future validation rather than a mandate for immediate practice change.

Another finding of interest was the interaction between autonomic vulnerability and rcSO_2_ decrease. One possible explanation, supported indirectly by physiological studies linking autonomic circulatory control, syncope or orthostatic intolerance, and cerebral perfusion or oxygenation [10], is that autonomic dysfunction may impair vasomotor responsiveness, blood pressure buffering, and cerebral autoregulatory reserve; in such patients, a given degree of intraoperative cerebral oxygen desaturation could reflect a greater physiologic burden than in patients with preserved autonomic compensation. However, this interpretation remains speculative and cannot be confirmed from the present observational data. The interaction was observed on the multiplicative scale in the primary analysis and with the stricter phenotype definition but was borderline in the screening-positive-only analysis. Our results are therefore hypothesis-generating and consistent with—but do not establish—the possibility that autonomic vulnerability may represent a susceptibility modifier for the association between intraoperative cerebral desaturation and POD. These interaction estimates should be used primarily to inform future prospective sample-size planning and mechanistic studies rather than to support confirmatory clinical inference.

The present results are broadly consistent with previous studies linking low perioperative cerebral oxygenation to delirium or adverse neurocognitive recovery [5,6,7,8,9,19,20], while extending this literature by incorporating a clinically defined autonomic vulnerability phenotype. Previous investigations have often focused on desaturation alone, without considering whether patient-level physiologic reserve modifies the observed association between desaturation and outcome [5,6,7,8,9]. The hypothesis that autonomic vulnerability may compound perioperative cerebral susceptibility is biologically plausible from the mechanistic literature linking autonomic circulatory failure, syncope, cerebral perfusion, and cerebral oxygenation [10], but this evidence should not be interpreted as direct proof of a perioperative AVP–POD causal pathway. The pathophysiological basis for POD is further supported by the neuroinflammatory and neurotransmitter-imbalance framework of acute brain failure [21], and the present study follows the consensus nomenclature recommendations for cognitive change associated with anaesthesia and surgery [22]. Age and dementia were also independently associated with POD in the conditional logistic regression model, which is clinically coherent because both variables index lower cognitive reserve and reduced resilience to perioperative physiologic stress [1,3,21,22,23]. Specifically, dementia (OR 2.80) was the strongest non-rcSO_2_ covariate retained in the model, consistent with reduced cholinergic reserve, altered blood–brain barrier integrity, and pre-existing neuroinflammatory priming that lower the threshold for acute postoperative cognitive decompensation [21]; the per-year age increment (OR 1.05), although modest, reflects cumulative age-related neurovascular and autoregulatory changes that compound the cerebral oxygenation effects observed here [1,3]. Their persistence after matching indicates that autonomic and cerebral oxygenation variables should be interpreted as additive risk markers superimposed on established geriatric neurocognitive vulnerability, not as replacements for conventional delirium risk assessment. The propensity score methodology applied in the present study aimed to reduce confounding by measured baseline covariates. The full-cohort IPTW estimates were approximately 8–9% lower than the matched-cohort estimates (AVP: OR 1.94 vs. 2.12; rcSO_2_ decrease ≥20%: OR 2.28 vs. 2.45), supporting directional robustness but also suggesting that residual confounding, common-support selection, or matching-related enrichment of older and more comorbid patients may have modestly inflated the matched estimates. By integrating preoperative vulnerability with intraoperative cerebral oxygenation, the current study offers a cautious association-based framework for interpreting POD risk and generating hypotheses for targeted monitoring strategies, although this framework requires prospective validation before clinical adoption.

This study has some limitations. First, the retrospective single-center design limits causal inference and may restrict external validity. Second, the autonomic vulnerability phenotype was defined pragmatically from documented clinical features rather than formal autonomic function testing, which may have introduced misclassification; however, a sensitivity analysis using a stricter definition produced consistent results. The composite AVP was retained as the primary exposure because its individual components were too sparse for adequately powered separate primary models and because they converge clinically on impaired cardiovascular autonomic regulation. Nevertheless, the composite pools neuropathic, orthostatic, syncopal, and chronotropic features with different hemodynamic mechanisms, and some components, particularly orthostatic hypotension and chronotropic incompetence, overlap mechanistically with intraoperative hemodynamic instability. This pathway overlap means that the AVP–rcSO_2_–POD relationship cannot be cleanly separated into independent causal and mediating pathways in this retrospective design. Third, delirium ascertainment relied on a combination of formal screening and chart review, and nearly one-third of POD cases were identified by chart review alone. This ascertainment heterogeneity may have influenced the primary findings, as the sensitivity analysis restricted to chart-reviewed POD yielded wide confidence intervals and nonsignificant results, likely reflecting both lower statistical power (n = 13 events) and potential differences in detection sensitivity. Chart-review-only cases may also represent a phenotypically different group of delirium episodes, including hypoactive, less severe, or shorter-duration episodes; consequently, pooling them with screening-positive cases increases outcome sensitivity but reduces phenotypic homogeneity. Fourth, selection into the analytic cohort depended on the availability of both intraoperative rcSO_2_ monitoring and postoperative delirium assessment; exclusion of patients lacking these data may have introduced selection bias. Fifth, residual confounding cannot be excluded despite propensity score matching and matched-cohort regression analysis. Sixth, the additive interaction measures (RERI, AP, SI) were calculated from odds ratios derived from conditional logistic regression; because the outcome exceeded 10% incidence in some subgroups, these estimates may overstate the magnitude of additive interaction on the risk scale. Seventh, the limited number of POD events (n = 41) relative to the number of model covariates constrains statistical power for interaction and subgroup analyses, and the results of secondary and stratified analyses should be interpreted with appropriate caution. Specifically, intraoperative hypotension episodes, depth of anesthesia (e.g., processed electroencephalogram-derived indices such as bispectral index), postoperative pain management and opioid consumption, perioperative benzodiazepine use, and postoperative intensive care unit admission were not systematically available for all patients and may represent unmeasured or incompletely measured confounders. Although preoperative cognitive impairment and frailty-related variables were included when available, standardized assessments (e.g., Mini-Mental State Examination, Mini-Cog, Montreal Cognitive Assessment, and Clinical Frailty Scale) were not uniformly performed, which may have resulted in incomplete adjustment for these important delirium risk factors. To address the limited events-per-variable ratio (approximately 5–6), Firth’s penalized logistic regression was performed as a supplementary analysis; the penalized odds ratios were within 8% of the primary conditional logistic regression estimates, suggesting that overfitting did not materially influence the results [24]. Bootstrap internal validation (1000 resamples) produced an optimism-corrected C-statistic of 0.76, indicating acceptable discrimination without substantial overfitting. Eighth, multiple secondary outcomes (n = 8) and sensitivity analyses (n = 6) were evaluated without formal multiplicity adjustment; accordingly, secondary and sensitivity findings should be interpreted as exploratory and hypothesis-generating rather than confirmatory. Ninth, this study was conducted at a single tertiary academic hospital in South Korea, and the cohort consisted exclusively of elderly Korean patients undergoing non-cardiac, non-neurological surgery. The generalizability of these findings to Western populations, other ethnic groups, different healthcare systems, cardiac or neurosurgical patients, and younger patient cohorts remains to be established. Differences in the prevalence of autonomic dysfunction, delirium risk profiles, perioperative care practices, and delirium screening protocols across settings may influence the external applicability of these results. Tenth, intraoperative hypotension sits on the plausible causal pathway linking autonomic vulnerability to cerebral desaturation and POD, and our binary vasopressor-use indicator is an incomplete proxy. In the sensitivity analysis that added time-weighted MAP < 65 mmHg exposure (Supplementary Table S4, analysis 4), the rcSO_2_ and AVP point estimates attenuated by approximately 15–20% but remained directionally consistent; residual confounding by hypotension cannot be excluded and may account for part of the observed associations. Eleventh, 32% of POD cases were identified by chart review alone, and the sensitivity analysis restricted to chart-reviewed cases was null; the primary effect estimates are therefore driven by the screening-positive subset, and the primary analysis should be understood to aggregate cases of uncertain phenotypic homogeneity. Twelfth, the composite AVP exposure pools clinically heterogeneous components (neuropathic, orthostatic, syncopal, and chronotropic), and the extent to which any one component drives the overall phenotype–POD association is addressed descriptively in Supplementary Table S6 but cannot be resolved in this single-center dataset. Descriptive metabolic and medication variables, including HbA1c availability, polypharmacy, and anticholinergic burden, are summarized in Supplementary Table S7.

Future studies should prospectively validate the autonomic vulnerability phenotype, standardize delirium detection, and test whether interventions aimed at reducing or preventing cerebral desaturation modify postoperative neurocognitive outcomes, particularly in patients with autonomic vulnerability. Such studies should prespecify formal autonomic testing, standardized baseline cognitive screening, and time-weighted hemodynamic measures so that vulnerability, desaturation, and hypotension can be separated more clearly. External validation in multicenter cohorts will be necessary to confirm generalizability. If replicated, the present findings could support a perioperative strategy that combines preoperative vulnerability assessment with individualized interpretation of intraoperative cerebral oximetry. Such an integrated approach would complement established multicomponent frameworks for the prevention and management of delirium in hospitalized older adults [25].

## 5. Conclusions

In this older and more comorbid subset of elderly non-cardiac, non-neurological surgical patients, a preoperative autonomic vulnerability phenotype and intraoperative rcSO_2_ desaturation were each independently associated with postoperative delirium after propensity score matching. The primary outcome association was driven mainly by screening-positive POD cases, whereas chart-review-only cases yielded imprecise and nonsignificant estimates. The association between cerebral desaturation and POD appeared stronger in patients with autonomic vulnerability, but this interaction was not uniformly robust across sensitivity analyses and attenuated when intraoperative hypotension was added as a sensitivity covariate. The findings should therefore be regarded as hypothesis-generating associations that require external validation and prospective confirmation, particularly in cohorts that include more representative samples of the broader elderly surgical population and that capture time-weighted intraoperative hemodynamics.

## Figures and Tables

**Table 1:** Baseline characteristics in the propensity score–matched cohort.

Variable	Autonomic Vulnerability Phenotype (n = 98)	No Phenotype (n = 98)	SMD
Age, y	74.5 ± 6.2	74.1 ± 5.9	0.07
Male sex	51 (52.0)	49 (50.0)	0.04
BMI, kg/m^2^	23.6 ± 3.5	23.9 ± 3.7	0.08
ASA physical status III–IV	67 (68.4)	66 (67.3)	0.02
Charlson Comorbidity Index	4.8 ± 2.3	4.6 ± 2.1	0.09
Hypertension	70 (71.4)	68 (69.4)	0.04
Diabetes mellitus	46 (46.9)	44 (44.9)	0.04
Coronary artery disease	18 (18.4)	17 (17.3)	0.03
Depression	9 (9.2)	8 (8.2)	0.04
Chronic kidney disease	29 (29.6)	27 (27.6)	0.04
History of stroke	16 (16.3)	15 (15.3)	0.03
Preexisting dementia	12 (12.2)	11 (11.2)	0.03
Abdominal surgery	38 (38.8)	36 (36.7)	0.04
Orthopedic surgery	31 (31.6)	33 (33.7)	0.04
Emergency surgery	18 (18.4)	17 (17.3)	0.03
Surgery duration, min	182.3 ± 73.6	175.8 ± 68.7	0.08
Estimated blood loss, mL	249.6 (100.4–419.8)	229.7 (90.3–399.5)	0.06
Transfusion	22 (22.4)	20 (20.4)	0.05
Vasopressor use	61 (62.2)	59 (60.2)	0.04

Values are presented as mean ± SD, median (interquartile range), or n (%). Standardized mean differences (SMDs) are shown to assess post-matching balance; SMD < 0.10 was considered acceptable balance. Abbreviations: ASA, American Society of Anesthesiologists; BMI, body mass index; CCI, Charlson Comorbidity Index; SMD, standardized mean difference. Note: Coronary artery disease and depression, which were included in the propensity score model, have been added to Table 1 for completeness of covariate reporting.

**Table 2:** Primary outcome: postoperative delirium within 5 days according to autonomic vulnerability phenotype in the propensity score–matched cohort.

Outcome	AVP (+) (n = 98)	AVP (–) (n = 98)	ARD (95% CI)	NNH (95% CI)
POD (primary), n (%)	27 (27.6)	14 (14.3)	13.3 pp (2.8–23.8)	7.5 (4.2–35.7)
Screening-positive POD, n (%)	19 (19.4)	9 (9.2)	10.2 pp (1.4–19.0)	9.8 (5.3–71.4)
Chart-reviewed POD, n (%)	8 (8.2)	5 (5.1)	3.1 pp (−3.6–9.7)	—

POD was the primary outcome. Screening-positive POD was identified by formal delirium screening (CAM-ICU or CAM) positivity alone (secondary outcome 1). Chart-reviewed POD was identified by structured chart review or physician documentation alone (secondary outcome 2). The absolute risk difference (ARD) is expressed in percentage points (pp). NNH denotes number needed to harm. Dashes indicate that the NNH was not calculated because the ARD was not statistically significant. Abbreviations: AVP, autonomic vulnerability phenotype; ARD, absolute risk difference; CAM, Confusion Assessment Method; CAM-ICU, Confusion Assessment Method for the Intensive Care Unit; CI, confidence interval; NNH, number needed to harm; POD, postoperative delirium.

**Table 3:** Intraoperative rcSO_2_ variables in the propensity score–matched cohort according to postoperative delirium status.

Variable	POD (n = 41)	No POD (n = 155)	*p* Value
Baseline rcSO_2_ (%)	61.8 ± 7.2	66.5 ± 6.1	<0.001
Intraoperative nadir rcSO_2_ (%)	47.5 ± 8.1	54.9 ± 6.8	<0.001
Relative rcSO_2_ decrease, %	21.2 ± 9.1	16.5 ± 7.8	0.004
rcSO_2_ decrease ≥20%	27 (65.9)	53 (34.2)	<0.001
Duration rcSO_2_ < 80% of baseline, min	18.7 (8.6–37.3)	7.2 (1.3–14.8)	<0.001
Area under the threshold < 80% (%·min)	157.6 (67.8–324.9)	51.8 (4.7–118.3)	<0.001

Data are presented as mean ± SD, median (interquartile range), or n (%). *p* values were derived from unpaired comparisons (independent-samples *t*-test or Mann–Whitney U test for continuous variables; chi-squared or Fisher’s exact test for categorical variables) within the propensity score–matched cohort. Because the original matching was performed on autonomic vulnerability status, patients regrouped by POD status do not preserve the matched-pair structure; therefore, unpaired tests were used for this descriptive comparison. Abbreviations: POD, postoperative delirium; rcSO_2_, regional cerebral oxygen saturation.

**Table 4:** Conditional logistic regression analysis for the primary outcome (postoperative delirium) in the propensity score–matched cohort.

Variable	OR	95% CI	*p* Value
Autonomic vulnerability phenotype	2.12	1.18–3.82	0.012
rcSO_2_ decrease ≥20%	2.45	1.31–4.58	0.005
Duration rcSO_2_ < 80% of baseline (per min)	1.02	1.01–1.04	0.008
Age (per year)	1.05	1.01–1.10	0.021
Dementia	2.80	1.30–6.05	0.008
Surgery duration (per 10 min)	1.04	0.99–1.08	0.077

Conditional logistic regression was used in the propensity score–matched cohort (98 pairs). The model included the autonomic vulnerability phenotype, rcSO_2_ decrease ≥20% (binary), and duration of rcSO_2_ < 80% of baseline as primary predictors, with additional adjustment for age, dementia, and surgery duration (the last included as an intraoperative covariate not part of the propensity score model). A multiplicative interaction between the autonomic vulnerability phenotype and rcSO_2_ decrease ≥20% was observed (interaction OR 2.10, *p* = 0.032). The same adjustment set (age, dementia, and surgery duration) was used consistently across the primary model and all supplementary and sensitivity analyses (Supplementary Tables S2–S4). Abbreviations: CI, confidence interval; OR, odds ratio; rcSO_2_, regional cerebral oxygen saturation.

## Data Availability

The data supporting the findings of this study are available from the corresponding authors upon reasonable request.

## Supplementary Materials

The following supporting information can be downloaded at: https://www.mdpi.com/article/10.3390/medicina62061065/s1, Figure S1: Directed acyclic graph; Figure S2: Love plot; Table S1: Spearman correlation matrix among intraoperative rcSO_2_-derived variables; Table S2: Stratified analysis according to autonomic vulnerability status; Table S3: Supplementary conditional logistic regression models using individual rcSO_2_-derived variables; Table S4: Sensitivity analyses for postoperative delirium; Table S5: Comparison of baseline characteristics between matched and unmatched patients; Table S6: Component-level breakdown of the autonomic vulnerability phenotype; Table S7: Preoperative metabolic and medication descriptors.

## Abbreviations

The following abbreviations are used in this manuscript:

AP	attributable proportion due to interaction
ARD	absolute risk difference
ASA	American Society of Anesthesiologists
AVP	autonomic vulnerability phenotype
BMI	body mass index
CAM	Confusion Assessment Method
CAM-ICU	Confusion Assessment Method for the Intensive Care Unit
CCI	Charlson Comorbidity Index
CI	confidence interval
HbA1c	glycated hemoglobin A1c
IPTW	inverse probability of treatment weighting
IQR	interquartile range
MAP	mean arterial pressure
NIRS	near-infrared spectroscopy
NNH	number needed to harm
OR	odds ratio
POD	postoperative delirium
rcSO_2_	regional cerebral oxygen saturation
RERI	relative excess risk due to interaction
SD	standard deviation
SI	synergy index
SMD	standardized mean difference
STROBE	Strengthening the Reporting of Observational Studies in Epidemiology
