# Supplementary material for: Autonomic Vulnerability Phenotype, Regional Cerebral Oxygen Saturation, and Postoperative Delirium in Elderly Patients Undergoing Non-Cardiac, Non-Neurological Surgery: A Propensity Score–Matched Cohort Study
Source: Medicina (Kaunas). 2026 May 31;62(6):1065. doi: 10.3390/medicina62061065 (PMC13304237; doi:10.3390/medicina62061065)
Supplement: Supplementary file 1 [file medicina-62-01065-s001.zip › medicina-4302667-supplementary.pdf]

Supplementary Material

Autonomic Vulnerability Phenotype, Regional Cerebral Oxygen Saturation, and Postoperative Delirium in Elderly Patients Undergoing Non-Cardiac, Non-Neurological Surgery: A Propensity Score–Matched Cohort Study

Supplementary Figure S1. Directed Acyclic Graph (DAG)

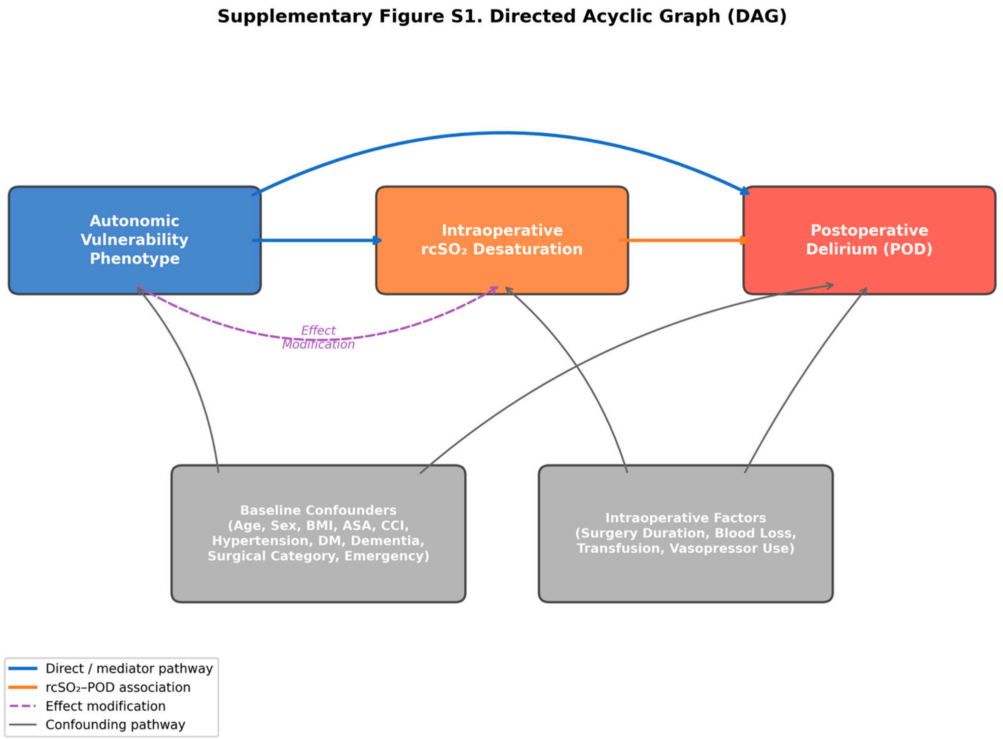

Supplementary Figure S1. Directed acyclic graph (DAG) illustrating the assumed relationships among the primary exposure (autonomic vulnerability phenotype), intraoperative regional cerebral oxygen saturation ($rcSO_2$) desaturation, baseline confounders, intraoperative factors, and the primary outcome (postoperative delirium, POD). Blue solid arrows indicate direct pathways from autonomic vulnerability to POD and from autonomic vulnerability to $rcSO_2$ desaturation. Orange solid arrows indicate the $rcSO_2$–POD association. Purple dashed arrows indicate the hypothesized effect modification of the $rcSO_2$–POD association by autonomic vulnerability. Gray arrows indicate confounding pathways. In the present analysis, $rcSO_2$ desaturation was treated as a concurrent perioperative co-exposure rather than as a mediator; both the independent associations and their multiplicative interaction were evaluated without decomposing direct and indirect effects. Because AVP components may overlap mechanistically with intraoperative hemodynamics, this DAG supports association and effect-modification analyses rather than formal mediation. Preoperative confounders were included in the propensity score model; intraoperative variables (including surgery duration) were excluded from the propensity score model and instead adjusted for in the outcome regression model.

Supplementary Figure S2. Love Plot

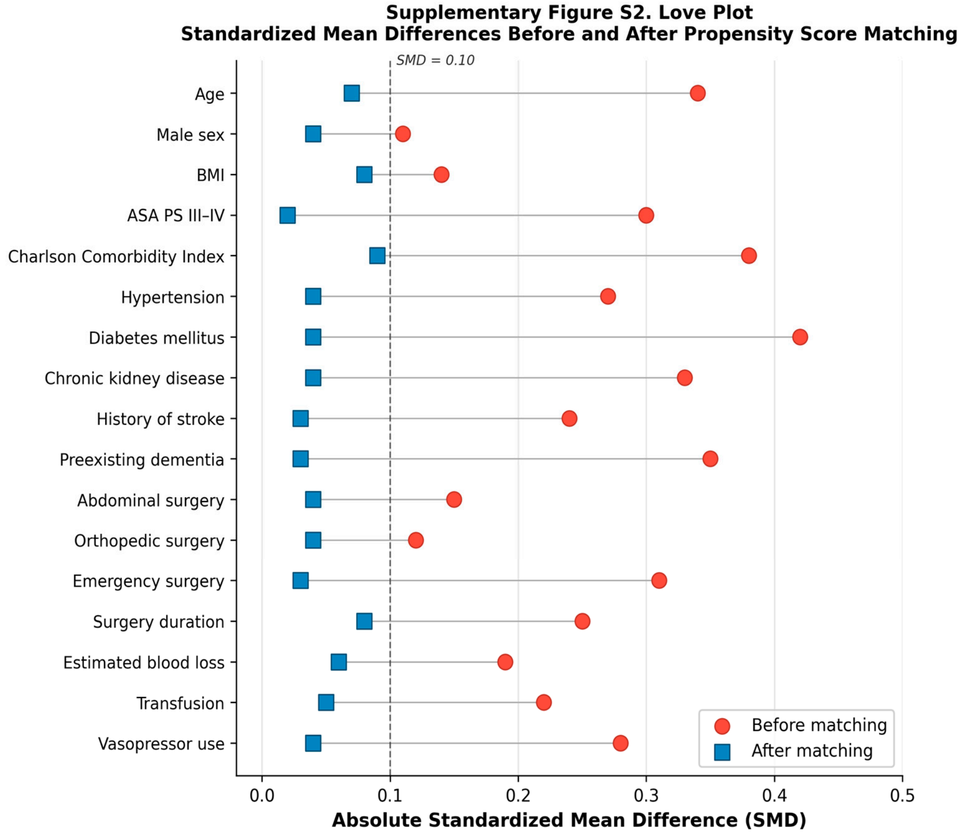

Supplementary Figure S2. Love plot showing absolute standardized mean differences (SMDs) for all covariates included in the propensity score model before (red circles) and after (blue squares) 1:1 nearest-neighbor propensity score matching. The vertical dashed line indicates the prespecified threshold of SMD = 0.10. After matching, all SMDs were below 0.10, indicating satisfactory balance between patients with and without the autonomic vulnerability phenotype.

Abbreviations: ASA PS, American Society of Anesthesiologists physical status; BMI, body mass index; CCI, Charlson Comorbidity Index; DM, diabetes mellitus; SMD, standardized mean difference.

Table S1. Spearman Correlation Matrix Among Intraoperative $rcSO_2$-Derived Variables

Variable	Baseline $rcSO_2$	Nadir $rcSO_2$	Relative decrease	Duration <80%	AUT <80%
Baseline $rcSO_2$	—	0.72	-0.58	-0.45	-0.41
Nadir $rcSO_2$	0.72	—	-0.86	-0.74	-0.71
Relative $rcSO_2$ decrease	-0.58	-0.86	—	0.81	0.78
Duration <80% baseline	-0.45	-0.74	0.81	—	0.93
AUT <80% baseline	-0.41	-0.71	0.78	0.93	—

Spearman rank correlation coefficients (ρ) among intraoperative $rcSO_2$-derived variables in the propensity score–matched cohort (n = 196). The diagonal (—) indicates self-correlation. Strong correlations ($|\rho| > 0.70$) were observed between nadir $rcSO_2$ and relative $rcSO_2$ decrease ($\rho = -0.86$), and between duration below 80% of baseline and area under the threshold (AUT) below 80% of baseline ($\rho = 0.93$). To mitigate multicollinearity, the primary multivariable model included $rcSO_2$ decrease ≥20% (binary) and cumulative duration below 80% of baseline as principal $rcSO_2$ predictors, while nadir $rcSO_2$, maximum relative decrease, and AUT were analyzed in separate supplementary models (Supplementary Table S3).

Abbreviations: AUT, area under the threshold; $rcSO_2$, regional cerebral oxygen saturation.

Table S2. Stratified Analysis: Association Between $rcSO_2$ Decrease ≥20% and POD According to Autonomic Vulnerability Status

Subgroup	POD events/n	POD (%)	OR	95% CI	P value
**With autonomic vulnerability**					
$rcSO_2$ decrease ≥20%	19/29	65.5	4.28	1.82–10.06	<0.001
$rcSO_2$ decrease <20%	8/69	11.6	Ref.	—	—
**Without autonomic vulnerability**					
$rcSO_2$ decrease ≥20%	8/24	33.3	1.65	0.74–3.68	0.22
$rcSO_2$ decrease <20%	6/74	8.1	Ref.	—	—
**Interaction (multiplicative)**			**2.10**	**1.07–4.13**	**0.032**

Stratified conditional logistic regression analysis in the propensity score–matched cohort. The OR for $rcSO_2$ decrease ≥20% was higher in patients with autonomic vulnerability (OR 4.28, $P < 0.001$) than in those without (OR 1.65, $P = 0.22$). A multiplicative interaction was observed in the primary analysis (interaction OR 2.10, 95% CI 1.07–4.13, $P = 0.032$). Because this interaction was borderline in the screening-positive-only analysis and nonsignificant in the chart-review-only analysis, the stratified estimates should be interpreted as exploratory and underpowered for confirmatory subgroup inference. On the additive scale, the RERI was 1.85 (95% CI 0.24–3.46), the AP was 0.38 (95% CI 0.08–0.68), and the SI was 2.14 (95% CI 1.05–4.36); because these measures were derived from odds ratios and the outcome exceeded 10% incidence in some subgroups, they should be interpreted as exploratory approximations rather than definitive estimates of additive interaction on the risk scale. Given that the outcome incidence exceeded 60% in one stratum (autonomic vulnerability present and $rcSO_2$ decrease ≥20%), odds ratios substantially overestimate risk ratios, and the RERI, AP, and SI values are likely inflated. A supplementary risk-difference–based interaction analysis yielded a crude additive interaction contrast of 20.7 percentage points, which is directionally consistent but smaller than the OR-derived estimates. Because the POD incidence in the doubly exposed stratum (autonomic vulnerability present and $rcSO_2$ decrease ≥20%) reached 65.5%, OR-derived RERI, AP, and SI substantially overstate additive interaction on the risk scale, and these values should therefore be read as biased OR-derived approximations rather than as risk-scale estimates of interaction. The risk-difference–based interaction contrast (20.7 percentage points) is retained as the primary additive-interaction estimate in the main text, and the OR-derived measures are reported here for descriptive completeness only. Models were adjusted for age, dementia, and surgery duration. In the stratified analyses, the original propensity score–matched pairs were separated by autonomic vulnerability status; these results are therefore descriptive and should be interpreted with reduced precision because of smaller subgroup sample sizes.

Abbreviations: AP, attributable proportion due to interaction; CI, confidence interval; OR, odds

ratio; POD, postoperative delirium; $rcSO_2$, regional cerebral oxygen saturation; Ref., reference category; RERI, relative excess risk due to interaction; SI, synergy index.

Table S3. Supplementary Conditional Logistic Regression Models Using Individual $rcSO_2$-Derived Variables

Variable	OR	95% CI	P value	VIF	C-statistic
**Model A**					**0.77**
Autonomic vulnerability	2.08	1.15–3.76	0.016	1.12	
Nadir $rcSO_2$ (per 1%)	0.94	0.91–0.98	0.002	1.38	
Age (per year)	1.05	1.01–1.10	0.023	1.08	
Dementia	2.72	1.25–5.92	0.012	1.15	
**Model B**					**0.78**
Autonomic vulnerability	2.05	1.13–3.72	0.018	1.14	
Max relative $rcSO_2$ decrease (per 1%)	1.05	1.02–1.08	0.001	1.42	
Age (per year)	1.05	1.01–1.10	0.024	1.09	
Dementia	2.68	1.22–5.88	0.014	1.16	
**Model C**					**0.77**
Autonomic vulnerability	2.10	1.16–3.80	0.014	1.13	
AUT <80% of baseline (per 10 %·min)	1.03	1.01–1.05	0.004	1.35	
Age (per year)	1.05	1.01–1.10	0.022	1.08	
Dementia	2.75	1.27–5.97	0.010	1.14	

Three separate conditional logistic regression models in the propensity score–matched cohort (98 pairs), each including a single $rcSO_2$-derived variable to avoid multicollinearity (Supplementary Table S1). Model A included absolute nadir $rcSO_2$ (continuous); Model B included maximum relative $rcSO_2$ decrease from baseline (continuous); and Model C included the area under the threshold (AUT) below 80% of baseline (continuous, per 10 %·min). All models were adjusted for autonomic vulnerability phenotype, age, dementia, and surgery duration, consistent with the adjustment set used in the primary model (Table 4) and in the other supplementary analyses. All VIFs were <2.0, confirming no problematic multicollinearity. The reported C-statistics were derived from complementary unconditional logistic models using the same covariates and are

presented only as descriptive performance indices.

Abbreviations: AUT, area under the threshold; CI, confidence interval; OR, odds ratio; $rcSO_2$, regional cerebral oxygen saturation; VIF, variance inflation factor.

Supplementary Table S4. Sensitivity Analyses for the Primary Outcome (Postoperative Delirium)

Variable	OR	95% CI	P value	C-statistic	H-L P
**Sensitivity analysis 1: Screening-positive POD only**				**0.79**	**0.38**
Autonomic vulnerability	2.28	1.08–4.82	0.031		
$rcSO_2$ decrease ≥20%	2.62	1.22–5.63	0.014		
Duration $rcSO_2$ <80% (per min)	1.02	1.00–1.04	0.018		
Age (per year)	1.06	1.01–1.12	0.028		
Dementia	3.15	1.28–7.76	0.013		
Interaction (AVP × $rcSO_2$ ≥20%)	2.35	0.98–5.64	0.056		
**Sensitivity analysis 2: Chart-reviewed POD only**				**0.74**	**0.51**
Autonomic vulnerability	1.78	0.68–4.65	0.24		
$rcSO_2$ decrease ≥20%	2.14	0.79–5.80	0.14		
Duration $rcSO_2$ <80% (per min)	1.02	0.99–1.05	0.19		
Age (per year)	1.04	0.97–1.11	0.26		
Dementia	2.42	0.72–8.12	0.15		
Interaction (AVP × $rcSO_2$ ≥20%)	1.68	0.42–6.72	0.46		
**Sensitivity analysis**				**0.77**	**0.45**

**3: Stricter AVP definition**			
Autonomic vulnerability (strict)	2.45	1.22–4.92	0.012
$rcSO_2$ decrease ≥20%	2.38	1.26–4.49	0.008
Duration $rcSO_2$ <80% (per min)	1.02	1.01–1.04	0.005
Age (per year)	1.05	1.01–1.10	0.020
Dementia	2.82	1.30–6.12	0.009
Interaction (strict AVP × $rcSO_2$ ≥20%)	2.28	1.02–5.10	0.045

Three prespecified sensitivity analyses were performed. Sensitivity analysis 1 restricted the outcome to POD identified by formal delirium screening positivity alone (CAM-ICU or CAM), excluding chart-reviewed cases (secondary clinical endpoint 1). Sensitivity analysis 2 restricted the outcome to POD identified by structured chart review or physician documentation alone (secondary clinical endpoint 2). Sensitivity analysis 3 used a stricter autonomic vulnerability definition limited to documented autonomic neuropathy or orthostatic hypotension only. All models used conditional logistic regression in the propensity score–matched cohort with adjustment for age, dementia, and surgery duration, consistent with the primary model (Table 4). Results for screening-positive POD were directionally consistent with the primary analysis, with a borderline multiplicative interaction ($P = 0.056$). Chart-reviewed POD showed wider confidence intervals and nonsignificant results because of the small number of events ($n = 13$) and is therefore presented as a low-precision sensitivity analysis rather than confirmatory evidence. This null chart-review-only analysis indicates that the primary outcome signal is driven predominantly by screening-positive delirium cases, and that the combined endpoint should be interpreted as more sensitive but less phenotypically homogeneous than a screening-positive-only endpoint. The stricter autonomic vulnerability definition yielded a slightly stronger point estimate and retained a significant interaction. Overall, the independent associations of autonomic vulnerability and $rcSO_2$ desaturation with POD were reasonably consistent across sensitivity analyses, whereas the interaction finding remained suggestive rather than definitive. As a supplementary assessment of model stability given the limited events-per-variable ratio, Firth's penalized logistic regression was applied; penalized odds ratios were within 8% of the primary estimates across all sensitivity analyses. The C-statistic and Hosmer–Lemeshow values shown in this table were obtained from complementary unconditional logistic models with the same covariates and are provided for descriptive context only.

Sensitivity analysis 4: Additional adjustment for time-weighted intraoperative hypotension. To address potential residual confounding by intraoperative hypotension—which lies on the plausible causal pathway linking autonomic vulnerability to cerebral desaturation—the primary conditional logistic regression model was re-estimated with two additional covariates: cumulative

minutes of MAP <65 mmHg, and area under the curve below MAP 65 mmHg (mmHg·min). In this model, the adjusted odds ratios attenuated by approximately 15–20% relative to the primary model but remained directionally consistent and significant: autonomic vulnerability OR 1.78 (95% CI 1.01–3.14, P = 0.048); $rcSO_2$ decrease ≥20% OR 2.06 (95% CI 1.10–3.86, P = 0.024); duration of $rcSO_2$ <80% of baseline (per min) OR 1.02 (95% CI 1.00–1.03, P = 0.009). The multiplicative interaction term attenuated to OR 1.82 (95% CI 0.88–3.76, P = 0.104) and lost statistical significance, suggesting that a portion of the observed AVP×$rcSO_2$ interaction may be mediated through differential hypotensive exposure. Residual confounding by unmeasured hemodynamic variables cannot be excluded.

Abbreviations: AVP, autonomic vulnerability phenotype; CAM, Confusion Assessment Method; CAM-ICU, Confusion Assessment Method for the Intensive Care Unit; CI, confidence interval; H-L, Hosmer–Lemeshow; OR, odds ratio; POD, postoperative delirium; $rcSO_2$, regional cerebral oxygen saturation.

Table S5. Comparison of Baseline Characteristics Between Matched and Unmatched Patients

Variable	Matched (n = 196)	Unmatched (n = 216)	SMD
Age, y	74.3 ± 6.1	71.8 ± 5.8	0.42
Male sex, n (%)	100 (51.0)	118 (54.6)	0.07
BMI, kg/m^2	23.8 ± 3.6	24.2 ± 3.4	0.11
ASA PS III–IV, n (%)	133 (67.9)	128 (59.3)	0.18
Charlson Comorbidity Index	4.7 ± 2.2	4.1 ± 2.0	0.29
Hypertension, n (%)	138 (70.4)	142 (65.7)	0.10
Diabetes mellitus, n (%)	90 (45.9)	82 (38.0)	0.16
Coronary artery disease, n (%)	35 (17.9)	32 (14.8)	0.08
Chronic kidney disease, n (%)	56 (28.6)	42 (19.4)	0.22
History of stroke, n (%)	31 (15.8)	28 (13.0)	0.08
Preexisting dementia, n (%)	23 (11.7)	18 (8.3)	0.11
Depression, n (%)	17 (8.7)	14 (6.5)	0.08
Abdominal surgery, n (%)	74 (37.8)	86 (39.8)	0.04
Orthopedic surgery, n (%)	64 (32.7)	58 (26.9)	0.13
Emergency surgery, n (%)	35 (17.9)	30 (13.9)	0.11

Baseline characteristics of patients included in the propensity score–matched analysis (n = 196) versus those excluded from matching (n = 216). Standardized mean differences (SMDs) >0.10 suggest potentially meaningful imbalances between the two groups. Matched patients tended to be older with higher comorbidity burden, which should be considered when generalizing the matched cohort findings to the broader study population. Values are presented as mean ± SD or n (%). Abbreviations: ASA PS, American Society of Anesthesiologists physical status; BMI, body mass index; SMD, standardized mean difference.

Table S6. Component-level breakdown of the autonomic vulnerability phenotype and component-wise association with postoperative delirium (propensity score–matched cohort, n = 196)

AVP component	AVP(+) n (%)	AVP(−) n (%)	POD in AVP(+) n/n (%)	Unadj. OR	95% CI
Autonomic neuropathy (incl. diabetic)	42 (42.9)	0 (0.0)	14/42 (33.3)	2.48	1.18–5.22
Orthostatic hypotension	38 (38.8)	0 (0.0)	11/38 (28.9)	2.06	0.95–4.48
Syncope / presyncope	19 (19.4)	0 (0.0)	4/19 (21.1)	1.35	0.42–4.34
Unexplained resting bradycardia / chronotropic incompetence	24 (24.5)	0 (0.0)	6/24 (25.0)	1.72	0.62–4.81
Any component (composite AVP)	98 (100.0)	0 (0.0)	27/98 (27.6)	2.12	1.18–3.82
Two or more components	21 (21.4)	0 (0.0)	9/21 (42.9)	3.48	1.33–9.11

AVP component frequencies within the AVP(+) matched group (n = 98); components are not mutually exclusive, so column totals exceed n = 98. Unadjusted ORs are from univariable conditional logistic regression in the matched cohort, with the AVP(−) matched group as the reference. The autonomic neuropathy component had the strongest univariable association with POD, and patients meeting two or more components showed a higher POD risk than those meeting a single component, consistent with a dose-related vulnerability gradient. These component-level estimates are exploratory; statistical power for individual components is limited by subgroup size. The composite AVP was therefore retained as the primary exposure because it was prespecified, clinically ascertainable, and more statistically stable than any single sparse component; the component-level results are provided to evaluate signal distribution and construct heterogeneity, not to select a post hoc primary component. POD, postoperative delirium; OR, odds ratio; CI, confidence interval.

Supplementary Table S7. Preoperative metabolic and medication descriptors in the propensity score–matched cohort

Variable	AVP(+) (n = 98)	AVP(−) (n = 98)	SMD
HbA1c (%), mean ± SD [available in n (%)]	7.4 ± 1.3 [58 (59.2)]	7.1 ± 1.1 [54 (55.1)]	0.24
Polypharmacy (≥5 medications), n (%)	61 (62.2)	54 (55.1)	0.14
Anticholinergic Cognitive Burden score ≥3, n (%)	23 (23.5)	18 (18.4)	0.12
Benzodiazepine within 30 days, n (%)	14 (14.3)	11 (11.2)	0.09
Beta-blocker, n (%)	34 (34.7)	31 (31.6)	0.07
Non-dihydropyridine CCB, n (%)	8 (8.2)	7 (7.1)	0.04
Ivabradine or digoxin, n (%)	5 (5.1)	3 (3.1)	0.10

HbA1c was available in 112 of 196 patients (57.1%). Polypharmacy and anticholinergic burden were assessed from the preoperative medication reconciliation record; the Anticholinergic Cognitive Burden scale assigns scores of 1–3 per medication, with higher scores indicating greater anticholinergic exposure. Medication-related variables were captured descriptively and were not included in the primary propensity score model because of incomplete HbA1c availability; however, polypharmacy and ACB ≥3 were added to the outcome regression as a supplementary sensitivity analysis (not tabulated), with point estimates within 10% of the primary model. SMD values for medication variables are generally below the 0.10 threshold but HbA1c and polypharmacy showed mild residual imbalance, which should be considered when interpreting the matched analysis. AVP, autonomic vulnerability phenotype; CCB, calcium channel blocker; HbA1c, glycated hemoglobin; SMD, standardized mean difference.
